# Improvement of mood and sleep alterations in posttraumatic stress disorder patients by eye movement desensitization and reprocessing

**DOI:** 10.3389/fnbeh.2014.00209

**Published:** 2014-06-10

**Authors:** Mara R. Raboni, Fabiana F. D. Alonso, Sergio Tufik, Deborah Suchecki

**Affiliations:** ^1^Group of Studies on the Neurobiology of Stress and Stress-Related Disorders, Departamento de Psicobiologia, Universidade Federal de São PauloSão Paulo, Brazil; ^2^Instituto do Sono, Associação Fundo de Incentivo à PesquisaSão Paulo, Brazil; ^3^Sleep Division, Departamento de Psicobiologia, Universidade Federal de São PauloSão Paulo, Brazil

**Keywords:** Posttraumatic stress disorder, EMDR, psychotherapy, depression, anxiety, sleep fragmentation

## Abstract

Posttraumatic stress disorder (PTSD) patients exhibit depressive and anxiety symptoms, in addition to nightmares, which interfere with sleep continuity. Pharmacologic treatment of these sleep problems improves PTSD symptoms, but very few studies have used psychotherapeutic interventions to treat PTSD and examined their effects on sleep quality. Therefore, in the present study, we sought to investigate the effects of Eye Movement Desensitization Reprocessing therapy on indices of mood, anxiety, subjective, and objective sleep. The sample was composed of 11 healthy controls and 13 PTSD patients that were victims of assault and/or kidnapping. All participants were assessed before, and 1 day after, the end of treatment for depressive and anxiety profile, general well-being and subjective sleep by filling out specific questionnaires. In addition, objective sleep patterns were evaluated by polysomnographic recording. Healthy volunteers were submitted to the therapy for three weekly sessions, whereas PTSD patients underwent five sessions, on average. Before treatment, PTSD patients exhibited high levels of anxiety and depression, poor quality of life and poor sleep, assessed both subjectively and objectively; the latter was reflected by increased time of waking after sleep onset. After completion of treatment, patients exhibited improvement in depression and anxiety symptoms, and in quality of life; with indices that were no longer different from control volunteers. Moreover, these patients showed more consolidated sleep, with reduction of time spent awake after sleep onset. In conclusion, Eye Movement Desensitization and Reprocessing was an effective treatment of PTSD patients and improved the associated sleep and psychological symptoms.

## Introduction

Posttraumatic stress disorder (PTSD) is the only psychiatric disorder in which a psychosocial stressor is the explicit etiologic factor (Olff et al., 2005). More than two thirds of the world's population are exposed to traumatic events at least once in their life, but only a small portion develops PTSD (Kessler et al., 1995; Breslau et al., 1998; Creamer et al., 2001; Norris et al., 2001), with a fourfold higher prevalence in women than in men (Stein et al., 1997). In addition, a high prevalence of co-morbidity with panic, agoraphobia, obsessive-compulsive disorder, major depression, somatization, and drug abuse has been reported (DSM-IV, 1994). This variability depends on individual characteristics, such as personality, perception of the traumatic event and coping strategies (Olff et al., 2005).

According to the DSM-IV, the chronic form of PTSD is characterized by the presence of symptoms, minimally after 3 months of the traumatic event, and the most significant ones include re-experiencing the traumatic event, with recurrent and intrusive recollections (flashbacks) and/or distressing dreams of the event, physiological reactivity upon exposure to internal or external cues that resemble the event or exposure to situational reminders, persistent avoidance of trauma-related stimuli, restricted range of affect, symptoms of increased arousal, including hypervigilance, and exaggerated startle response (DSM-IV, 1994). PTSD patients present with reduced parasympathetic tonus, on the one hand, and augmented sympathetic activity (Cohen et al., 1997; Rothbaum et al., 2001; Blechert et al., 2007) on the other, which may explain, at least in part, the arousal and hypervigilance characteristic of this condition (Mellman et al., 1995c).

Currently, sleep alterations, particularly those involving REM sleep (Mellman et al., 2002), such as increased REM density (Mellman et al., 1995a; Pillar et al., 2000) are considered hallmarks of PTSD (Hefez et al., 1987; Ross et al., 1989; Spoormaker and Montgomery, 2008). Co-morbidity between PTSD and sleep complaints reaches impressive rates (Kato et al., 1996; Ohayon and Shapiro, 2000; Pillar et al., 2000), and this is particularly relevant for a disorder that involves stressful situations, since sleep has been proposed to be a natural form of resetting the activity of stress response systems, whereas sleep deprivation or poor sleep augments the activity of these systems (Suchecki et al., 2012). Moreover, recent findings point to the possibility that sleep disorders may not only be a consequence (Spoormaker and Montgomery, 2008), but also a precipitating factor of PTSD (Bryant et al., 2010; Van Liempt, 2012). Therefore, PTSD patients may be under the control of a vicious circle that involves poor sleep quality leading to exacerbated stress response, which results in worsening of sleep.

One way of breaking this vicious circle is to treat one of the conditions and examine the outcome on the other. On the one hand, both pharmacological and psychological treatments for sleep disorders have been shown to improve PTSD symptoms (Krakow et al., 2001a,b, 2002; Germain et al., 2012). Conversely, treatment of PTSD, by different kinds of psychotherapy has yielded controversial results. Reports indicate that although patients exhibit improvement of PTSD symptoms, sleep problems, such as insomnia (Zayfert and Deviva, 2004) and poor sleep quality (Galovski et al., 2009) still persist. Importantly, these studies have only assessed subjective sleep. In a recent study, an 8-week period of Cognitive Behavioral Therapy (CBT) for insomnia was offered to PTSD patients, producing subjective and objective improvement of sleep, depressive symptoms and social adjustment (Talbot et al., 2014). To the best of our knowledge, no study had evaluated the effects of Eye Movement Desensitization and Reprocessing (EMDR®) on subjective sleep complaints and objective sleep pattern in PTSD patients. We chose to employ EMDR given the evidence that this treatment, together with trauma focused CBT, is effective in reducing clinician assessed PTSD symptoms (Van Etten and Taylor, 1998; Bisson and Andrew, 2007; Bisson et al., 2007; Rhudy et al., 2010). This psychotherapy involves alternated bilateral sensorial stimulation at the same time that the traumatic event is being processed (Shapiro, 1996). According to Francine Shapiro, who developed the therapy, “Processing (or reprocessing) is thus defined as the forging of the associations required for learning to take place as the information pertaining to the traumatic event is adaptively resolved” (Shapiro, 1999), e.g., when the patient ceases to present emotional reactions while retrieving the traumatic situation.

The present study was, therefore, carried out to test the hypothesis that treatment of PTSD with EMDR would improve the patients' psychological and general well being, and sleep quality measured both objectively and subjectively.

## Methods

### Subjects

Patients (PTSD group) and healthy volunteers (Control, CTL—group) were recruited from May 10, 2004 to July 2, 2008, by newspaper, TV and radio announcements, followed by referral from selected participants. All subjects were informed of the study objectives and procedures, as well as the possible benefits of EMDR. Those interested in participating in the study signed an informed consent form and all procedures were approved by the Ethics Research Committee of Universidade Federal de São Paulo, in accordance with the Declaration of Helsinki (CEP # 354/03). Because this study commenced before the requirement of registration in Clinical Trials, it was only registered last year (http://www.ensaiosclinicos.gov.br/rg/edit/1456/), and approval is still pending.

Healthy control volunteers were enrolled to match the patients, as close as possible, for age, sex, and educational level. No financial incentive was provided to increase adherence to the protocol. All participants were capable of understanding the objectives and procedures involved in this study.

### Inclusion criteria

The traumatic event was restricted to assault and/or kidnapping which took place within 3 months to 5 years prior to enrollment in the study. Participants were of both sexes, 24–40 years old and were, at least, high school graduates, BMI < 30 kg/m^2^ and living in São Paulo City. This time frame was chosen based on DSM-IV criteria for chronic PTSD symptoms (duration for 3 months or more) (DSM-IV, 1994).

Participants answered questions regarding the traumatic event and how long ago it occurred, as well as demographic data, such as age, years of school, sex, and occupation. All participants were interviewed to confirm whether they fulfilled the required criteria to participate in the study (as described above) and eligible ones were treated by the psychologist in charge (MRR) in the clinical center of the Department of Psychobiology. All participants were submitted to a psychiatric evaluation for confirmation of PTSD diagnosis and absence of any psychiatric conditions for the CTL group. Psychiatric interview consisted of application of the Structured Clinical Interview for DSM-IV Axis I Disorders (SCID-I), translated and validated to Brazilian Portuguese (Del Ben et al., 2001).

Initially, 24 PTSD patients were enrolled in the study; however, 11 were not included in the final analyses because they: (1) reported illnesses diagnosed after the onset of treatment (hyperthyroidism); (2) were victims of more than one traumatic event different from the specific one (sexual assault, job accident, domestic violence); (3) missed EMDR sessions due to work commitments; (4) experienced stressful life events, such as divorce, home or town moving and work change. Even though these patients were excluded from the study, they were offered to continue the treatment, but only five did so. The final sample size, therefore, included twenty-four subjects, 11 healthy volunteers (CTL group) and 13 PTSD patients (PTSD group).

### Exclusion criteria

The following were used as exclusion criteria: (1) use of any medication that could interfere with sleep architecture; (2) use of psychotropic drugs; (3) use of any medication that could increase rapid eye movements; (4) clinical and neurologic disorders; (5) past history of neurologic, endocrine or hepatic disease; (6) dissociative disorders or psychosis; and (7) history of sleep disorder (either assessed objectively or reported by the patient) previous to the traumatic event.

### Questionnaires

Volunteers and patients filled out the questionnaires listed below, before and after the end of treatment. All questionnaires were validated for Brazilian Portuguese.

- *Questionnaire of social-economic status* (ABIPEME, 2003), was used to assess social-economic status according to the Brazilian Association of Market Research Institutes. This is an instrument that classifies the subject in social categories according to his/her economic status and level of education. The five social classes range from A to E, class E being the lowest.
- *Impact of Event Scale* (Horowitz et al., 1979): was used to measure the impact of the traumatic event that contributed to triggering PTSD. It identifies intrusive and avoidance symptoms following the traumatic event. It was also used to assess the effects of reprocessing during each session of EMDR.
- *Beck Depression Inventory* (Beck et al., 1961): is a 21-item self-reported inventory designed to measure the severity of depression. Scores can range from 0 to 63, and are classified as follows: minimal, 0–11; mild, 12–19; moderate, 20–35; and severe, 36–63.
- *State Trait Anxiety Inventory* (Spielberger et al., 1970): is composed of two scales: state-anxiety and trait-anxiety and each scale contains 20 items, in which the subject marks how he/she usually feels (trait) or how he/she feels at that time (state).
- *Recovery-Stress-Questionnaire – RESTQ-48* (Kellmann and Kallus, 2001): was used to assess the level of stress with the purpose of monitoring the recovery capacity from psychological and physical stressors in the last 7 days. It is composed of 12 general scales used to gather information about the emotional, personal, social, and work routines of the subject. Among the questions, those related to sleep are: (19) I feel fresh, satisfied and relaxed; (27) I slept well; (36) I had a restless sleep; (46) My sleep was easily disturbed. Subjects attributed a score from 0 to 6 to each question of the questionnaire and a median was calculated. All aspects were assessed together and a final score was reached.
- *Pittsburgh Sleep Quality Index* (Buysse et al., 1989), was used to assess the patients and volunteers subjective sleep quality and their sleep habits. It has seven components—subjective quality of sleep, latency to sleep, sleep duration, sleep efficiency, sleep disorders, use of sleeping medication and daytime sleepiness—which result in a score of global subjective sleep quality. The global score is determined by the sum of all components, varying from 0 to 3, where 3 is the most negative in a Likert-type scale. The score ranges from 0 to 21, those higher than five being an indication of bad sleep quality.
- *SF-36 Life Quality* (Ware and Sherbourne, 1992): is composed of 11 questions and 36 items that encompass eight dimensions represented by functional capacity, physical aspects, pain, general health status, vitality, social aspects, emotional aspects, mental health, and one comparative question about current and past (last year) health perception. In each dimension, score varies from 0 (worst) to 100 (best).
- *Scale of social adjustment* (Weissman and Bothwell, 1976): This scale is composed of 54 questions, 42 of which are related to work and the subject must answer six out of 18 questions regarding his/her main occupation. The items assess aspects of performance and quality of interpersonal relationships and feelings of personal fulfillment in the last 2 weeks. The lower the score, the better the social adjustment.
- *Subjective Units of Distress Scale* (SUD): This scale is used to assess the patient's discomfort at the beginning and end of each EMDR session. The patient attributes a score from 0 to 10, where 0 represents no distress and 10 represent the highest distress possible.

### Objective sleep assessment

Objective sleep evaluation was carried out, individually, by whole-night polysomnography in the Sleep Institute, by technical specialized personnel, on three nights: Night 1: Adaptation to the laboratory and to recording cables and equipment; Night 2: Basal polysomnography, before the onset of EMDR treatment (PSG 1) and Night 3: Post-treatment recording, performed on the night following the end of treatment (PSG 2). Polysomnography was performed with three channels of electroencephalogram (EEG: C3-A2; C4-A1; O2-A1), two channels of electrooculogram (EOG), one submental and one tibial electromyogram, and one electrocardiogram. Sleep scoring was performed according to Rechstchaffen and Kales (Rechtschaffen and Kales, 1968), using 30 s epochs. Wakening and respiratory events and periodic leg movements were considered according to the guidelines of the American Sleep Disorders Association and the American Association of Sleep Medicine (AASM, 1999; ASDA, 2003).

The following parameters were obtained by the polysomnography (PSG): (1) Sleep latency (min): interval between the onset of the recording and the first epoch of stage 2; (2) Latency to REM sleep (min): time interval between sleep onset and the first REM episode; (3) Total sleep time (TST - min): time interval between sleep onset and offset, excluding all periods of awakening; (4) Sleep efficiency (%): Percentage of TST during total recording time; (5) Sleep stages (N1, N2, N3, and REM): expressed as percentage of TST; (6) REM density: visual counting of rapid eye movements (above 25 μV) during each period of REM sleep; and (7) Time of waking after sleep onset (WASO, in min): sum of all periods of waking after sleep initiation. Women were sleep recorded between the 6th and 25th day of the menstrual cycle. In order to examine the sequence of sleep stages, as an additional evaluation of sleep continuity, polysomnograms were inspected individually and transitions between REM sleep and waking, REM sleep and N1, and REM sleep and N2 were counted.

An accredited polysomnography technician, blind to the group condition, analyzed the sleep recordings.

### EMDR procedure

EMDR treatment was applied according to the protocol established by Shapiro (Shapiro, 1995) and following the guidelines established by Foa and Meadows (Foa and Meadows, 1997). The individual session began with a brief explanation of the technique. Subjects of the PTSD group were asked to recall a trauma-related scene or image, whereas Control subjects were asked to recall an aversive or highly unpleasant situation. Each volunteer was asked to identify a physical feeling or sensation, negative and positive beliefs related to the trauma. PTSD patients were asked to assess his/her level of discomfort in a Likert-type scale from 0 to 10, the negative belief in the SUD scale and the desired positive belief of this event, in a 1-7 scale, where 1 represents completely false and 7, completely true. After this initial evaluation the bilateral stimulation began (guided eye movement through horizontal motion of the therapist's arm or alternated tapping on the patient's knees) and after approximately 12 motions the patients were asked to report new scenes and reevaluate physical sensations and feelings. At the end of the session, the therapist (MRR) checked the patient's condition and if complete sensitization of the material (SUD < 1) had occurred. If it had not, the session was wrapped up with a “safe place” relaxation technique. It is worth mentioning that during EMDR, the traumatic memory, as well as feelings, emotions, ideas, images, and behaviors related to this memory emerge, so the patient can face and process the trauma, under a safe situation. Therefore, re-experiencing the trauma is extremely intense to patients, regardless of how long he/she was exposed to the trauma.

Each weekly session lasted approximately 90 min, and subjects attended a minimum of three to a maximum of 10 sessions, depending on their self-report improvement during the reprocessing and resolution of the traumatic experience, which were classified as: negative emotions (fear, insecurity, despair, sadness, anguish, impotence, disgust, tension, stress, guilt, discomfort), positive emotions (tranquility, relaxation, relief, self-control, caution, happiness, overcoming, well-being, hope, freedom, feelings of safety, lightness) and anger. Heart rate was recorded in PTSD patients, during each therapy session by a Polar™ monitor connected to a chest belt, every 10 min, together with a report of the predominant feeling among the abovementioned ones.

**Figure 1:**
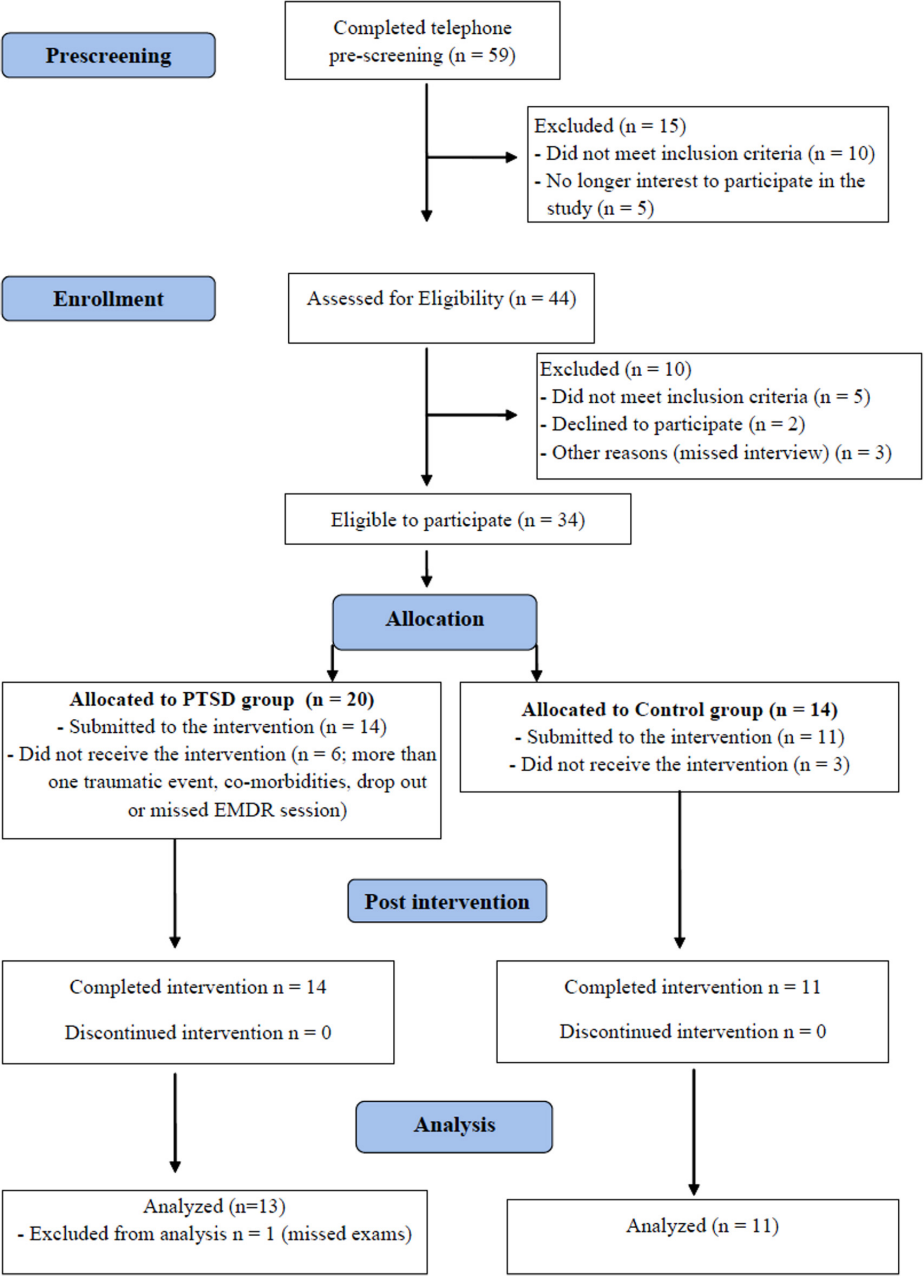
**Flow chart indicating the total number of individuals assessed, recruited and treated by EMDR (CONSORT)**.

Subjects in PTSD and Control groups came in for a first visit, when they were informed about the procedures, signed the informed consent and filled out the psychological profile questionnaires. They were scheduled for two consecutive nights of PSG before beginning EMDR sessions. On the night after the last EMDR session, the subjects underwent a final PSG and filled out the questionnaires once again. The whole treatment lasted two and a half months, on average. Figure 1 shows the flow chart diagram of the present study.

### Statistical analysis

For psychological variables, between group comparisons (CTL × PTSD in each phase) were carried out by the Mann-Whitney *U* test, whereas within group comparisons (before x after treatment), by the Wilcoxon paired test. Objective sleep parameters were analyzed by a Two-Way repeated ANOVA with group (CTL, PTSD) and time-point (repeated measure: before, after treatment) as main factors. Heart rate was analyzed by a One-Way ANOVA for repeated measures, with time-point (sessions 1 through 5) as the main factor. *Post-hoc* analysis was done by the Newman-Keuls Test and the level of significance adopted was *p* < 0.05; the software used was *Statistica* v. 7.0 (Copyright© StatSoft, Inc.). Cohen's D effect size was calculated for all sleep parameters. This is an index that measures the magnitude of an effect and the calculator is available at http://www.uccs.edu/~faculty/lbecker/. Values of 0.5–0.8 represent moderate relevance, whereas those above 0.81, high relevance (http://www.uccs.edu/~faculty/lbecker/es.htm).

## Results

There were no differences between groups in regard to the ratio of women:men, age, socio-economic status and education (Table 1). Eleven PTSD patients dropped out of the study for several reasons, including: illnesses revealed after the onset of treatment; patients who reported having being exposed to more than one traumatic event; patients who missed more than one therapy session or had family problems that precluded their adherence to the treatment.

**Table 1:** **Demographic data of the participants, regarding sex, age, and years of education**.

**Groups**	**Sex**		**Age (y)**	**Education (y)**
	**Women**	**Men**		
CTL (*N* = 11)	8	3	29.0 ± 4.4	17.8 ± 1.3
PTSD (*N* = 13)	10	3	30.5 ± 5.2	15.5 ± 4.3

CTL, Control; PTSD, Post-traumatic stress disorder.

### Psychological well-being and objective sleep

Table 2 shows the results of psychological, stress perception and well-being assessments. Regarding the impact of the event, naturally, PTSD patients showed higher scores than CTL group (*p* < 0.002) before the beginning of treatment (*U* = 16.5; *p* < 0.002). These differences were no longer detectable after the treatment, but the comparison between before and after treatment showed that the impact of the event was reduced in all groups (*p* < 0.05).

**Table 2:** **Psychological, stress perception and quality of life of Control (CTL) and posttraumatic stress disorder (PTSD) patients, assessed by specific questionnaires**.

**Psychological aspects**	**Before treatment**		**After treatment**	
	**CTL (***N*** = **11**)**	**PTSD (***N*** = **13**)**	**CTL (***N*** = **11**)**	**TEPT (***N*** = **13**)**
Impact event scale	15.4±6.5	27.0±5.9^*^	10.1±8.1^¥^	7.8±6.8^¥^
BDI—depression	4.2±4.4	17.4±6.8^*^	2.2±2.4	4.0±4.6^¥^
STA!—state	33.4±8.6	45.5±9.1^*^	34.1±9.2	34.7±9.1^¥^
STAI—trait	34.1±6.9	54.1±10.0^*^	30.0±7.6	36.1±8.6^¥^
RESTQ—general stress	0.9±0.8	3.1±1.3^*^	0.6±0.7	1.0±0.7^¥^
RESTQ—emotional stress	1.2±0.8	3.5±1.3^*^	1.0±0.9	1.5±0.5^¥^
RESTQ—social stress	1.1±1.2	3.0±1.1^*^	0.8±1.1	1.2±1.0^¥^
RESTQ—fatigue	2.0±1.6	3.5±1.4^*^	1.9±1.5	1.4±1.2^¥^
RESTQ—general well-being	4.4±0.6	2.5±1.2^*^	4.8±0.7	4.6±0.9^¥^
SF36—quality of life	87.0±6.4	54.2±14.4^*^	89.0±9.7	85.7±8.0^¥^
Scale of social adjustment	1.52±0.3	2.24±0.5^*^	1.44±0.3	1.83±0.5^¥^

Values are presented as mean ± SD. Number of subjects per group is shown in parenthesis.

* Different from CTL group (Mann-Whitney U Test);

¥ Different from before treatment (Wilkoxon Rank Test).

Depression scores were also different between the groups before the onset of treatment (*U* = 5.5; *p* < 0.0005), with higher scores for PTSD patients than CTL subjects. After treatment, the groups were no longer different, and PTSD patients exhibited lower levels of depression than before treatment (*p* < 0.002).

Before treatment, the level of state-anxiety (*U* = 23.5; *p* < 0.006) was greater in PTSD than in CTL group, but this was no longer observed after the treatment. Regarding the level of trait-anxiety, PTSD patients showed higher scores than CTL group (*U* = 8.5; *p* < 0.0003). Again, trait-anxiety did not differ among the groups after the treatment, with PTSD patients exhibiting reduction of both state and trait anxiety, following treatment (*p* < 0.005 in both cases).

The results of perception of general stress showed differences between the groups before treatment (*U* = 10; *p* < 0.0005), in which PTSD group had higher scores than the CTL group, but this difference was no longer observed after EMDR. Regarding emotional stress, the results were similar to those of general stress (*U* = 6.0; *p* < 0.0002). Before treatment, a significant difference was observed in the social stress domain (*U* = 16.0; *p* < 0.002) and fatigue (*U* = 34.0; *p* < 0.04), with PTSD group showing higher scores than the CTL group in both domains. These differences were not observed after EMDR. In view of these results, the general well-being was different between the groups (*U* = 8.0; *p* < 0.0003), and PTSD patients exhibited the worst condition before treatment.

According to the SF-36 questionnaire, prior to treatment PTSD patients exhibited worse quality of life than the CTL group (*U* = 2.0; *p* < 0.0001). No differences among the groups were observed after the treatment.

The groups also differed in social adjustment, both before (*U* = 10.0; *p* < 0.0004) and after the treatment (*U* = 35.0; *p* < 0.04); PTSD patients exhibited higher scores than CTL subjects, e.g., poor social adjustment, (*p* < 0.0003), but after EMDR there was an improvement in this parameter for PTSD patients (*p* < 0.02).

**Figure 2:**
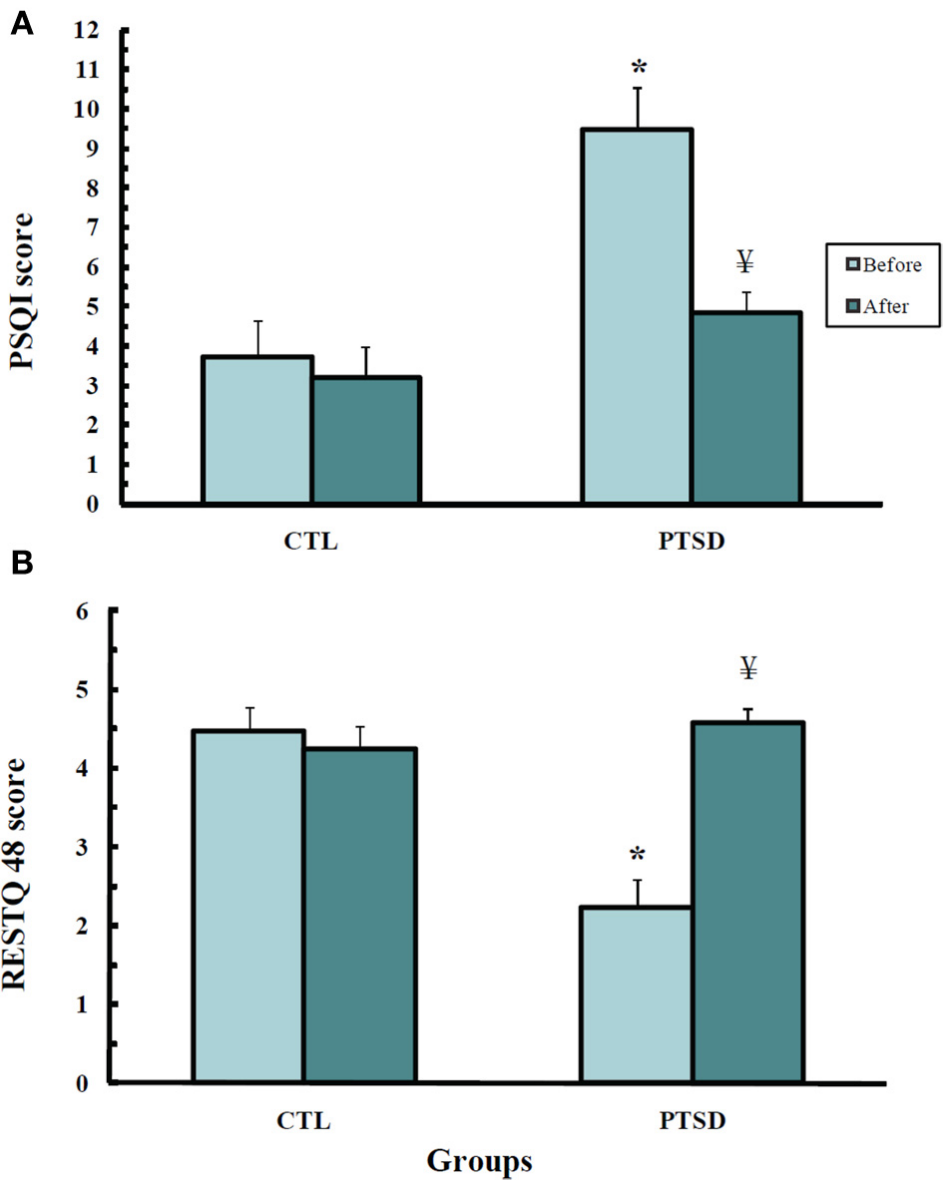
**Scores obtained from the Recovery-Stress-Questionnaire—RESTQ-48 (A) and the Pittsburgh Sleep Quality Index (B) of control (CTL, *N* = 11) and post-traumatic stress disorder (PTSD) patients (*N* = 13)**. Data were obtained before and after EMDR therapy. Values are expressed as mean ± s.e.m. ^*^Different from CTL group; ^¥^Different from before treatment.

Self-assessment of sleep quality in the RESTQ-48 questionnaire (Figure 2A) showed differences among the groups (*U* = 13.0; *p* < 0.0008); PTSD patients evaluated their sleep as being worse than the CTL (*p* < 0.001) group. After the treatment, the patients attributed an improvement to their sleep (*p* < 0.002).

A similar result was obtained with the Pittsburg Sleep Index Quality (Figure 2B) (*U* = 17.0; *p* < 0.002); before treatment PTSD patients self-rated their sleep quality as being worse than that of CTL subjects (*p* < 0.003). After the treatment, PTSD patients did not exhibit any difference from healthy volunteers.

### Sleep parameters

**Figure 3:**
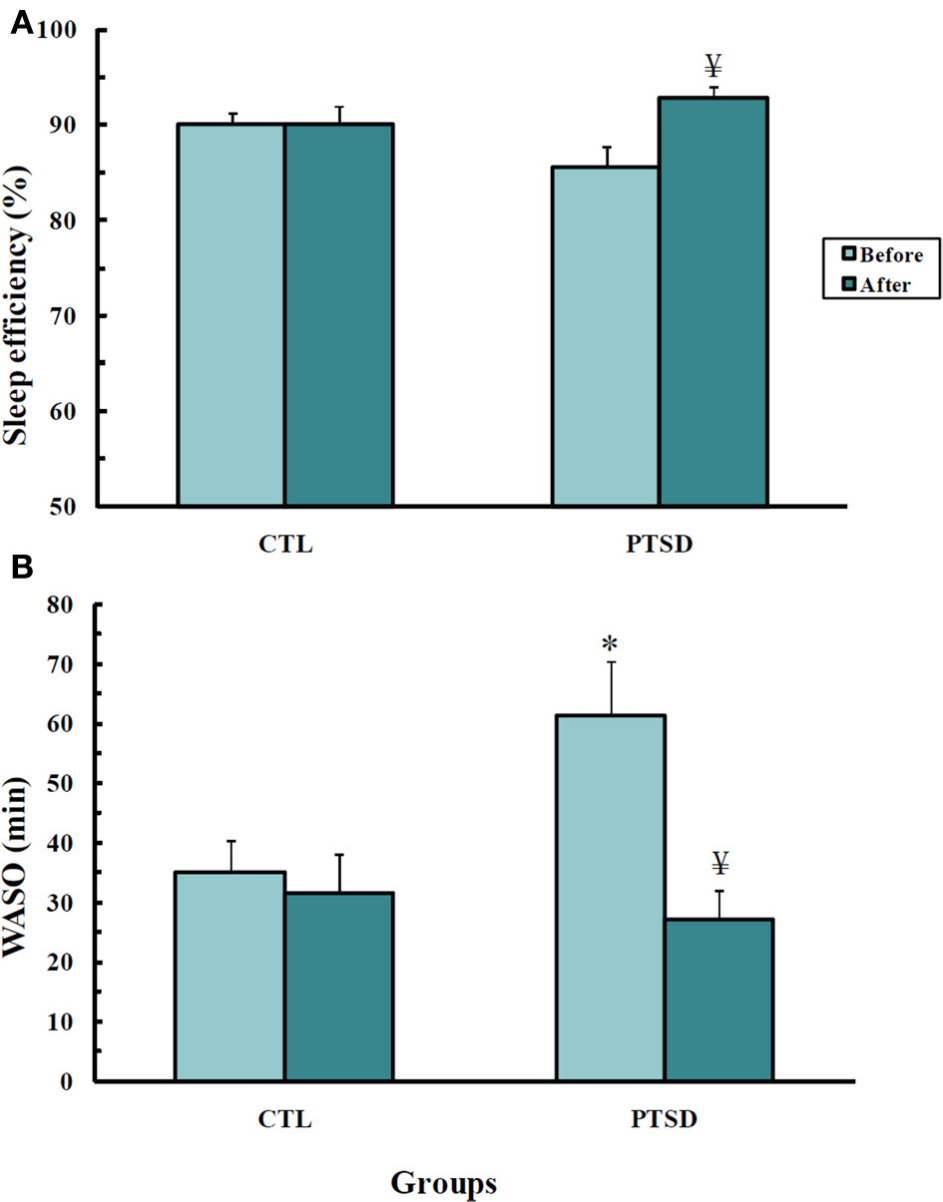
**Sleep efficiency (A) and waking after sleep onset (B), in min, of control (CTL, *N* = 11) and post-traumatic stress disorder (PTSD) patients (*N* = 13)**. Data were obtained before and after EMDR therapy. Values are expressed as mean ± s.e.m. ^*^Different from CTL group; ^¥^Different from before treatment.

Results obtained in the polysomnography are shown in Figure 3 and Table 3 (non-significant results). Although statistical differences were not detected by ANOVA for transitions from REM sleep to awakening and for REM density, the comparison between PTSD and CTL groups, before the onset of treatment, revealed an effect size of 0.65 and 1.05, respectively, indicating medium and high clinical relevance. These differences were no longer present after the treatment. Table 4 shows the results of effect size for all parameters.

**Table 3:** **Sleep parameters recorded before and 1 day after the end of EMDR therapy applied to control (CTL) and posttraumatic stress disorder (PTSD) patients**.

**Sleep parameters**	**Before**		**After**	
	**CTL (***N*** = **11**)**	**PTSD (***N*** = **13**)**	**CTL (***N*** = **11**)**	**PTSD (***N*** = **13**)**
Sleep latency (min)	10.3±6.1	8.3±4.3	13.5±15.6	5.8±7.1
REM sleep latency (min)	83.2±36.5	81.2±28.9	84.5±39.4	91.4±31.5
N1 (%)	2.7±2.4	3.9±4.3	1.9±1.2	2.8±1.7
N2 (%)	57.8±6	54.7±4.5	55.8±6.8	54.8±6.4
N3 (%)	21.0±6.0	22.2±6.3	22.2±7.8	21.7±8.1
Micro-arousals (n)	71.2±25.4	80.1±29.2	75.5±28.1	72.3±29.1
Total sleep time (min)	383.1±24.3	375.8±57.3	377.4±66.3	379.5±47.9
PLM (n/h)	0.6±2.2	0.6±2.1	0.6±2.0	0.7±2.1
AHI (n/h)	2.1±1.7	4.8±6.7	2.8±2.1	4.2±6.2
Transitions REM-waking	3.0±1.7	4.8±3.5	2.9±1.8	3.8±2.7
Transitions REM-N1	0.4±0.7	0.4±0.6	0.6±1.0	0.6±0.8
Transitions REM-N2	2.5±0.9	2.4±1.6	3.4±2.2	2.5±1.4
REM density^a^	34±18	62±33	29±21	26±17

Values are presented as mean ± SD. Number of subjects per group is shown in parenthesis.

a REM density was determined in 9 control subjects and 13 PTSD patients.

**Table 4:**
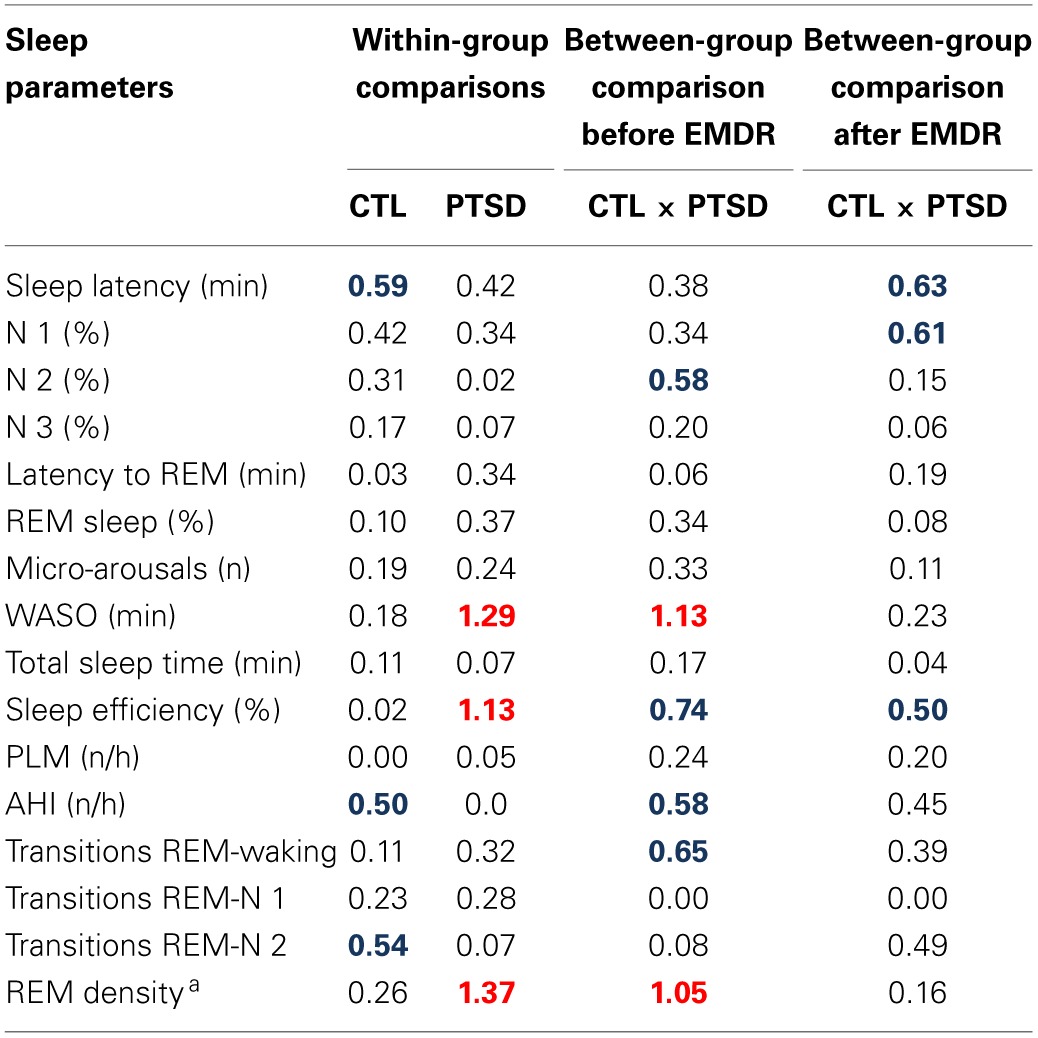
**Cohen D effect size was calculated for all sleep parameters**.

Comparisons were made within each group (before x after EMDR) and between groups in each time-point. Bold blue values represent moderate clinical relevance (values between 0.5 and 0.8), whereas those in red represent great clinical relevance (above 0.8).

^a^ REM density was determined in 9 control subjects and 13 PTSD patients. WASO, waking after sleep onset, PLM, periodic limb movements; AHI: apnea-hypopnea index.

### Sleep efficiency (figure 3A)

There was an interaction between group and time-point [*F*_(1, 22)_= 4.74; *p* = 0.04]. PTSD patients had greater sleep efficiency after EMDR than before the treatment (*p* < 0.03).

### Waking after sleep onset (WASO, figure 3B)

An interaction between group and time-point was detected [*F*_(1, 22)_= 6.51; *p* < 0.02]. *Post hoc* analysis showed that before the treatment PTSD patients spent more time awake than control subjects (*p* < 0.01); after the treatment these patients showed a reduction of this parameter (*p* < 0.003), being indistinguishable from controls.

**Figure 4:**
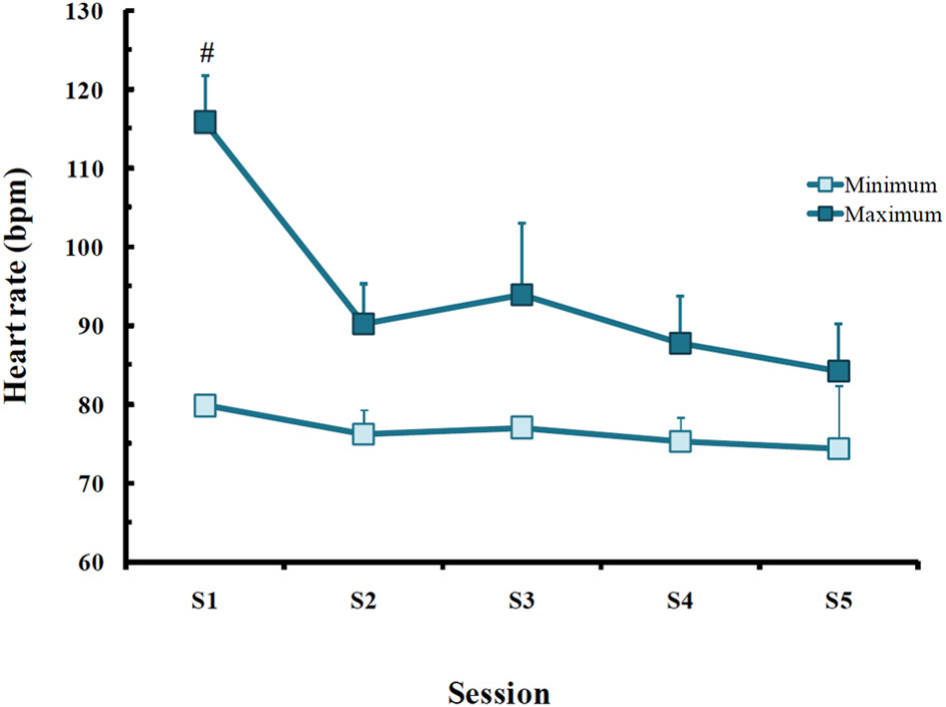
**Minimum and maximum heart rate (bpm) obtained in post-traumatic stress disorder (PTSD) patients in each EMDR session**. Values are expressed as mean ± *SD* of 13 patients. ^#^Different from all other sessions.

Throughout the treatment, there were differences only in maximum HR in PTSD patients [*F*_(1, 4)_ = 4.13; *p* < 0.02], with higher rate in the first session than in all others (*p* < 0.03; Figure 4).

## Discussion

The present study tested the hypothesis that treatment of PTSD would improve the general well-being and sleep quality of affected subjects. The results showed that, indeed, PTSD patients had a significant improvement in all psychological parameters evaluated. Interestingly, before treatment PTSD patients self-rated their sleep very poorly, but objective measurements showed that, in fact, the most remarkable sleep alteration was a longer WASO time than control volunteers.

The therapy chosen to treat PTSD symptoms helps patients to process the information about the traumatic event leading to a resolution and improvement of the condition (Levin et al., 1999) and its efficacy is attested by several studies using civilian populations with reports of improvement after a few sessions (Wilson et al., 1995; Rothbaum, 1997; Scheck et al., 1998; Bisson and Andrew, 2007). In the present study, patients required an average of 5 sessions to reprocess the traumatic memory. Regarding their psychological profile, there was a robust improvement in depression and anxiety scores, which were significantly higher before the onset of treatment and became comparable to CTL subjects at the end of treatment. It has been reported that PTSD patients present hyperactivity of the noradrenergic system during waking (Southwick et al., 1999) and sleep (Mellman et al., 1995b). Interestingly, our patients exhibited a reduction of maximum heart rate after the first EMDR session, and it has been shown in previous studies that EMDR causes a shift in the sympathetic/parasympathetic activity toward increased parasympathetic tonus, reflected by decreased heart rate, decreased skin galvanic resistance and increased fingertip temperature in PTSD patients (Dunn et al., 1996; Sack, 2007; Elofsson et al., 2008). Importantly, reduction of the sympathetic tonus may also be involved in extinction of fear memory that is observed with EMDR therapy. PTSD patients exhibit increased amygdala activation both at resting state (Rabinak et al., 2011) or after stimulus provocation (El Khoury-Malhame et al., 2011) and animal data show that increased noradrenergic activity is a major mediator of consolidation of fear memories by the amygdala (Roozendaal et al., 2009; Debiec et al., 2011). Therefore, reduced sympathetic activation may, at least in part, explain the improvements observed after EMDR. Recent data obtained from 10 PTSD patients submitted to EMDR while being recorded by electroencephalogram, showed that this therapy shifted the activation of limbic emotion-related structures to cortical regions involved with cognitive and associative processes, leading to improvement of the cognitive and sensorial processing of the traumatic event (Pagani et al., 2012). Only a few imaging studies have been carried out to evaluate neuroanatomical changes induced by EMDR. These studies report increased hippocampal volume in patients treated for eight [case-study (Letizia et al., 2007)] or 12 weeks [29 PTSD patients (Bossini et al., 2012)] and activation of the anterior cingulate gyrus and the left frontal lobe upon recall of the traumatic event [case-study, three EMDR sessions (Levin et al., 1999)]. Despite the small sample size, these results seem to be promising, given the role that these brain areas play in memory and emotion (Levin et al., 1999; Bush et al., 2000).

PTSD patients underestimate their sleep efficiency and overestimate sleep latency, but polysomnographic studies do not consistently show differences in sleep pattern between patients and healthy volunteers (Dagan et al., 1991; Hurwitz et al., 1998; Engdahl et al., 2000), suggesting that the patients' perception of sleep quality may be impaired. In our sample, a similar phenomenon was observed. Almost all sleep parameters were statistically indistinguishable between patients and controls; nonetheless, the effect size revealed that, indeed, PTSD patients presented more sleep disturbances than healthy volunteers, such as greater apnea/hypopnea index, more transitions from REM sleep to waking and greater REM density, in agreement with various previous reports (Ross et al., 1989, 1994; Breslau et al., 2004). Summation of these events may have led to the significantly longer waking time after sleep onset in PTSD patients observed before treatment. Studies concerning the sleep pattern of PTSD patients consensually report problems of sleep continuity (Mellman et al., 1995b; Yetkin et al., 2010; Capaldi et al., 2011) and EMDR was capable of restoring WASO to normal levels, as seen in healthy volunteers. Moreover, this therapy also increased sleep efficiency, resulting in a more consolidated sleep, although before the onset of treatment PTSD patients displayed similar values compared to control subjects. Interestingly, pre-clinical work has shown that certain forms of stress induce REM sleep rebound (Rampin et al., 1991; Palma et al., 2000; Sanford et al., 2010), a phenomenon thought to be important for recovery from the adverse situation. However, there are some features related to the stimulus and to the subject (anxiety level) that interfere with the sleep outcome (Suchecki et al., 2012). In our sample, PTSD patients had higher scores of both trait and state anxiety, and this could be a psychological disadvantage in stressful or traumatic situations, as shown by mice and rat strains that display more anxiety-like behavior and fail to exhibit the characteristic increase in REM sleep time after a mild stressor (Meerlo et al., 2001; Sanford et al., 2003; Tang et al., 2005). Although the experimental protocol used in the present study did not allow assessment of post-trauma sleep, Mellman et al. (2002) showed that consolidated periods of REM sleep within a month of a traumatic event lowered the probability of accident victims to develop PTSD.

Studies designed to investigate the effects of PTSD treatments on objective sleep parameters are scarce. These treatments usually are successful for PTSD, but residual insomnia is often a complaint (Zayfert and Deviva, 2004; Belleville et al., 2011). A recent study, carried out to treat residual insomnia in PTSD patients, compared the effects of a specific CBT protocol for insomnia with a waitlist non-treated group and showed improvements of insomnia and depression symptoms in treated, compared to non-treated patients, although both groups presented improvement of PTSD symptoms at the end of treatment period (Talbot et al., 2014). The present approach differed from Talbot's, because we intended to assess the efficacy of a treatment for PTSD on sleep parameters. Thus, we compared PTSD patients to healthy volunteers who were also submitted to the therapy for three reasons. First, we wanted to rule out the possibility of a placebo effect, which could have occurred just by the comforting feeling that patients experience for being taken care of. Second, we also wanted to make sure that possible sleep changes would not be due to habituation to sleep recording setting. Previous reports indicate that PTSD outpatients are more sensitive to sleeping in an unknown place (Woodward et al., 1996; Ross et al., 1999) (also known as first night effect), therefore, habituation to the sleep recording setting could have precluded the results. Third, we wanted to have reference values for the assessed parameters and not only compare the patients before and after the treatment, e.g., improvement of the symptoms might not mean return to control, healthy levels. The fact that EMDR induced changes only in PTSD patients and that they did not differ from controls at post-treatment time, indicated that this was a successful approach to test the proposed hypothesis of this study.

In conclusion, PTSD patients presented high levels of depression and anxiety before treatment, whereas, after the therapy all variables were indistinguishable from those of control subjects. Moreover, some sleep impairments were also improved by EMDR. Therefore, treatment of PTSD ameliorated the general well-being of the patients, but it is not possible, within the experimental design of this study, to establish cause-effect relationships.

### Limitations of the study

Various criteria were applied in order to gather a more homogenous sample of PTSD patients, such as restrictions to any previous therapy, to a single traumatic event, to the type of trauma and to the time span between the traumatic event and onset of EMDR to a maximum of 5 years. This strategy resulted in a small number of participants, which was worsened by the significant amount of drop-outs. Despite this drawback, we believe that such rigor added credibility to the results.

A second limitation of this study refers to the selection of one type of trauma, which hindered generalization of the effects of EMDR on the outcomes reported in the present study. Although this therapeutic approach has been used in several populations, this is the first study, to the best of our knowledge, to assess its effects on sleep. Therefore, the possibility of type of trauma-related biased effect exists, although this seems to be unlikely.

### Conflict of interest statement

The authors declare that the research was conducted in the absence of any commercial or financial relationships that could be construed as a potential conflict of interest.
